# Stiffness-mediated mesenchymal stem cell fate decision in 3D-bioprinted hydrogels

**DOI:** 10.1093/burnst/tkaa029

**Published:** 2020-07-27

**Authors:** Yufan Liu, Zhao Li, Jianjun Li, Siming Yang, Yijie Zhang, Bin Yao, Wei Song, Xiaobing Fu, Sha Huang

**Affiliations:** 1 Research Center for Tissue Repair and Regeneration affiliated to the Medical Innovation Research Department, PLA General Hospital and PLA Medical College, 28 Fu Xing Road, Beijing 100853, P. R. China; 2 PLA Key Laboratory of Tissue Repair and Regenerative Medicine and Beijing Key Research Laboratory of Skin Injury, Repair and Regeneration; Research Unit of Trauma Care, Tissue Repair and Regeneration, Chinese Academy of Medical Sciences, 2019RU051, 51 Fu Cheng Road, Beijing 100048, P. R. China

**Keywords:** mesenchymal stem cells, Stiffness, Adipogenesis, Osteogenesis, Differentiation, 3D-bioprinting

## Abstract

**Background:**

Hydrogels with tuneable mechanical properties are an attractive material platform for 3D bioprinting. Thus far, numerous studies have confirmed that the biophysical cues of hydrogels, such as stiffness, are known to have a profound impact on mesenchymal stem cell (MSC) differentiation; however, their differentiation potential within 3D-bioprinted hydrogels is not completely understood. Here, we propose a protocol for the exploration of how the stiffness of alginate–gelatin (Alg-Gel) composite hydrogels (the widely used bioink) affects the differentiation of MSCs in the presence or absence of differentiation inducing factors.

**Methods:**

Two types of Alg-Gel composite hydrogels (Young’s modulus: 50 kPa vs. 225 kPa) were bioprinted independently of porosity. Then, stiffness-induced biases towards adipogenic and osteogenic differentiation of the embedded MSCs were analysed by co-staining with alkaline phosphatase (ALP) and oil red O. The expression of specific markers at the gene level was detected after a 3-day culture.

**Results:**

Confocal microscopy indicated that all tested hydrogels supported MSC growth and viability during the culture period. Higher expression of adipogenic and osteogenic markers (ALP and lipoprotein lipase (LPL)) in stiffer 3D-bioprinted matrices demonstrated a more significant response of MSCs to stiffer hydrogels with respect to differentiation, which was more robust in differentiation-inducing medium. However, the LPL expression in stiffer 3D-bioprinted constructs was reduced at day 3 regardless of the presence of differentiation-inducing factors. Although MSCs embedded in softer hydrogels to some extent proceeded toward adipogenic and osteogenic lineages within a few days, their differentiation seemed to be slower and more limited. Interestingly, the hydrogel itself (without differentiation-inducing factors) exhibited a slight effect on whether MSCs differentiated towards an adipogenic or an osteogenic fate. Considering that the mechano-regulated protein Yes-associated protein (YAP) is involved in MSC fate decisions, we further found that inhibition of YAP significantly downregulated the expression of ALP and LPL in MSCs in stiffer constructs regardless of the induced growth factors present.

**Conclusions:**

These results demonstrate that the differentiation of MSCs in 3D-bioprinted matrices is dependent on hydrogel stiffness, which emphasizes the importance of biophysical cues as a determinant of cellular behaviour.

Highlights

- The stiffness was associated with the Alg-Gel concentrations.
- Bioprinted by stiffer Alg-Gel hydrogels can cause changes in MSC cell fate decision.
- The stiffness of Alg-Gel hydrogels may regulate the differentiation of MSC through YAP-related pathways

## Background

Advancements in 3D bioprinting technology have resulted in great potential for advancing regenerative tissue research [1–4]. Sodium alginate–gelatin (Alg-Gel) composite hydrogels with adjustable mechanical properties are an attractive bioink for bio-extrusion moulding because of their good cell compatibility, printability and structure maintenance in long-term culture [5–7]. Recently, it has been proposed that in addition to biological and structural cues, the pore size and porosity, and even the bioink itself, also regulate stem cell differentiation within Alg-Gel hydrogel-based bioink [5, 6, 8–14]. While the generation of 3D-bioprinted constructs using this widely available bioink has become an approach towards understanding how the matrix affects cell fate, the regulatory role of biophysical cues such as stiffness in 3D-bioprinted gels is still unclear. Thus far, many related studies have confirmed that stiffness is a key biophysical cue in cell fate decisions. Embryonic cardiomyocytes cultured on a stiffer matrix are similar to normal myocardium in terms of their differentiated state and beat, and it has been shown that neonatal cardiomyocytes cultured on a physiologically stiff matrix elongate with myofibril alignment [15–18]. A stiff extracellular matrix (ECM) composed of elevated-collagen levels could trigger cancer stem cell-like programming and metastatic dissemination of lung cancer *in vivo* [19]. Mammary gland progenitor cells that are cultured on a softer matrix tend to differentiate into luminal epithelial cells, while the same cells cultured on a stiffer matrix tend to differentiate into myoepithelial cells [20]. Furthermore, benign breast cells are transformed into malignant breast cancer cells when cultured on a stiffer matrix [21]. According to previous studies, mesenchymal stem cells (MSCs) in a softer matrix, tend to differentiate into adipocytes, whereas they tend to differentiate into osteoblasts in a stiffer matrix [22–25]. However, in most studies, the tremendous variation in porosity coupled with stiffness tuning also regulates stem cell differentiation. Therefore, it is critical to decouple stiffness and porosity of Alg-Gel hydrogel-based bioink to determine whether and how they regulate stem cell differentiation. Additionally, it is unclear whether the stiffness-mediated regulation that occurs in 3D-bioprinted gels is sufficient to induce MSC differentiation independently of osteogenic- and adipogenic-inducing medium (O/A medium). Thus, we constructed MSC-laden 3D-bioprinted matrices by modulating stiffness without altering the porosity of Alg-Gel composite hydrogels; we then analysed stiffness-induced biases towards adipogenic and osteogenic differentiation of embedded MSCs. To exclude the effects of inductive factors on MSC differentiation, we cultivated these 3D-bioprinted matrices in two types of media (Dulbecco’s modified Eagle’s medium (DMEM) and O/A medium). We showed that varying the stiffness did not significantly change the porosity of the Alg-Gel composite hydrogels, but changes in stiffness did influence whether the MSCs proceeded towards an osteogenic or adipogenic differentiation lineage. These effects were minimal without the inclusion of inductive factors that collectively regulate stem cell differentiation. Yes-associated protein (YAP)/tafazzin (TAZ) activation has recently been reported as the molecular mechanism by which the biophysical properties, such as stiffness, of bioprinted ECM direct the induction of MSC differentiation [26, 27]. Consequently, in this study, we further investigated whether YAP inhibition impacted the stiffness-mediated regulation of stem cell differentiation in 3D-bioprinted hydrogels.

## Methods

### Preparation of Alg-Gel composite hydrogels

The Alg-Gel composite hydrogels were prepared according to Table 1. Using the 1A3G group as an example, 1 g of sodium alginate (120–190 kDa, 39% guluronic acid, 180947-100G, Sigma, USA) and 3 g of gelatin (40–100 kDa, type B, G9382 Sigma, USA) were weighed on an electronic balance (JM-B2003, China). The weighed sodium alginate (Alg) and gelatin (Gel) were placed in a volumetric flask containing 100 mL of ultrapure water, which was fully stirred and evenly mixed. The Alg-Gel blend was maintained at 60°C for 12 h to allow the components to completely dissolve and was then pasteurized. After sterilization, the Alg-Gel composite hydrogels were sealed and stored at 4°C until subsequent experiments.

**Table 1:** Composition of alginate-gelatin (Alg-Gel) composite hydrogel in each group

Group	Sodium alginate (g)	Gelatin (g)	Ultrapure water (mL)
1A3G	1	3	100
2A5G	2	5	100
3A5G	3	5	100
3A8G	3	8	100
4A10G	4	10	100
5A10G	5	10	100

### Physical properties of Alg-Gel composite hydrogels

A compression test was used to measure the Young’s modulus of each group of Alg-Gel composite hydrogels [28]. The samples were formed into a cylinder with a height (*h*) of 20 mm and a diameter (*d*) of 20 mm. The samples were placed on a compression device (INSTRON Model 5567 machine), with a load of 100 N and a descending speed of 3 mm/min until the samples broke. Tests were repeated three times for each group to obtain the average values. The first 10% of the compression curve was selected, and the correlation curve was drawn with strain (*ε*) as the abscissa and stress (*σ*) as the ordinate. Equation (1) was used to calculate Young’s modulus, *E*:

(1) \begin{equation*} E=\sigma /\varepsilon \end{equation*}

where *σ* is the stress and *ε* is the strain.

The microstructures of Alg-Gel composite hydrogels were analysed by scanning electron microscope (SEM S-4800, HITACHI, Tokyo, Japan). Briefly, the samples were freeze-dried (Christ Alpha 2–4 LD Freeze Dryer) for 48 h and then sprayed with gold (20 nm, Edwards sputter coater).

The absolute ethanol displacement method [28, 29] was used to measure the porosity of the Alg-Gel composite hydrogels. First, 20 mL of Alg-Gel composite hydrogels were loaded into a 20 mL syringe, cooled and solidified at 4°C and then squeezed into a 50 mL centrifuge tube (Corning, USA) containing 40 mL of 2.5% CaCl_2_ solution for crosslinking. After the 2.5% CaCl_2_ was discarded, the samples were freeze-dried for 48 h, and then 45 mL of absolute ethanol (*V1*) was added after which the sample was maintained at 4°C for 48–36 h. Equation (2) was used to calculate the porosity,

(2) \begin{equation*} Porosity=\left(V1\hbox{--} V3\right)/\left(V2\hbox{--} V3\right)\times 100\% \end{equation*}

where *V1* is the volume of absolute ethanol, *V2* is the total volume after adding absolute ethanol and *V3* is the volume of absolute ethanol remaining after the sample was removed after immersion for 48–36 h. The calculations for each group were repeated three times to obtain the average values.

### Isolation and culture of mouse MSCs

Isolation and culture of mouse MSCs were performed according to previous studies [5, 6, 30]. One-week-old C57BL/6 mice were purchased from the SPF Laboratory Animal Center (Beijing, China). The mice were sacrificed and immersed in 75% alcohol for 10 min. First, the mouse’s feet and tail were removed, and then the skin on the mouse’s legs was cut. The femur and tibia of the mouse were separated and placed on a 100 mm cell culture dish (Corning, USA) containing complete MesenCult™ medium (mouse) (STEMCELL, Canada). Then the bones were crushed into small pieces using haemostatic forceps and then cut into 1 mm [3] fragments using ophthalmic scissors. Second, the medium was discarded and a type I collagenase solution (0.25% type I collagenase, 20% foetal bovine serum, phosphate buffer saline (PBS)) was added. The Petri dish was maintained at 37°C for 45–60 min and shaken every 5 min. Finally, after centrifugation at 300–400 g for 10 min, the primary MSCs were harvested and incubated with complete MesenCult™ Medium (Mouse) at 37°C and 5% CO_2_. The medium was changed after 72 h, and when passaged, the cells were digested in 0.05% trypsin and 0.02% ethylenediaminetetraacetic acid (EDTA, Gibco, Canada). All cell culture processes were completed under sterile conditions. For this experiment, second- to third-generation MSCs were used [31]. All animal experiments were performed in accordance with the guidelines of the Institutional Animal Care and Use Committee of Chinese PLA General Hospital (Beijing, China). All experimental protocols were approved by the Institutional Animal Care and Use Committee of Chinese PLA General Hospital (Beijing, China, approval number SCXK(BJ)2017–0001).

### Construction of MSC-laden 3D-bioprinted matrices

The1A3G and 4A10G hydrogels were sealed and pasteurized for sterilization. The MSC suspension (1 mL, 1.0 × 10 [7] cells) and 9 mL of the 1A3G or 4A10G hydrogel were thoroughly mixed and sealed in a sterile printing syringe. A bioprinting platform (Regenovo 3D Bio-printer, China) was used to construct the MSC-laden 3D-bioprinted matrices. After cooling at 4°C for 30 min (1A3G) or 15 min (4A10G), the sterile print syringe was mounted on the print arm (15°C) of the bioprinting platform, while a 60 mm Petri dish (Corning, America) was placed on the pre-cooled printing platform (4°C) containing the hydrogel-MSC mixture. The MSC-laden 3D-bioprinted matrix with a diameter of 30 mm, a thickness of 3 mm and pores 2.5 mm in diameter, was formed using a layer-by-layer pattern of a print nozzle with a diameter of 260 μm. The 3D-bioprinted matrices were immersed in sterile 2.5% CaCl_2_ solution for 10 min to cross-link the matrix. The cross-linked matrices were placed in a sterile incubator and cultured at 37°C and 5% CO_2_.

DMEM containing 10% foetal calf serum and O/A medium (MesenCult™ Osteogenic medium:MesenCult™ Adipogenic medium, 1:1 STEMCELL, Canada) were used to culture the matrices. The media were changed daily. The YAP inhibitors Verteporfin (VP, Selleck, China) and GW5074 (Selleck, China) were added to the media at working concentrations of 1 μM [dissolved in dimethyl sulfoxide (DMSO)] and 5 μM (dissolved in DMSO), respectively. DMSO (MP Biomedicals, USA) was used in the control group.

### Cell adhesion, extension and viability in 3D-bioprinted matrices

After the matrices were fixed in 4% paraformaldehyde solution for 20 min, they were embedded in optimal cutting temperature (O.C.T) (Sakura, America) and frozen at −25°C, after which the embedded matrices were cut into thin slices and pasted on adhesive slides. Firstly, the slides were subjected to antigen retrieval, after which the slides were permeabilized with 0.3% Triton X-100 (Sigma-Aldrich) for 10 min and incubated in 5% goat serum (Zsbio, China) in PBS for 1 h to block non-specific protein interactions. Second, the slides were incubated with an anti-β-actin mouse monoclonal antibody (AC-15) (1:200, ab6276, Abcam) and an anti-paxillin rabbit monoclonal antibody (Y113) (1:200, ab32084, Abcam) for 12–16 h at 4°C. Third, the slides were placed in a humidified chamber and incubated with conjugated AffiniPure goat anti-mouse (1:200, CoraLite488, SA00013–1) and conjugated goat anti-rabbit (1:200, CoraLite594, SA00013–4) antibodies for 2 h at room temperature in the dark. Nuclei were counterstained with DAPI Fluoromount-G (0100–20, Southern Biotech). A laser scanning confocal microscope (Leica, SP8 FALCON, Germany) was used to obtain fluorescence images.

A LIVE/DEAD® Viability/Cytotoxicity Kit (Invitrogen, USA) was used to stain the 3D-bioprinted matrices at days 1 and 3 after printing to analyse the viability of MSCs in the matrices. An inverted fluorescence microscope (Leica, BMI4000, Germany) was used to obtain fluorescence images.

### Real-time quantitative polymerase chain reaction analysis for the detection of the adipogenic and osteogenic differentiation of MSCs in the matrices

The cells obtained after dissolution [32] of the matrices were fully lysed in TRIzol (Invitrogen) and then transferred to 1.5 mL Eppendorf (EP) tubes with 1 mL per tube. Total RNA was extracted according to a previously published method [5, 6, 9, 30] and was reverse-transcribed with a PrimeScript™–RT reagent Kit (TaKaRa, China). cDNA was amplified using TB Green™–Premix Ex Taq™ II (TaKaRa, China). The QuantStudio 5 system (ThermoFisher, America) was used to analyse the polymerase chain reaction (PCR) data. All data were analysed using the *C(t)* value comparison method. The primers used are shown in Table 2.

**Table 2:** Primer sequences of *Lpl* and *Alp* genes

Primer	Sequence (Forward)	Sequence (Reverse)
*Lpl*	GGGAGTTTGGCTCCAGAGTTT	TGTGTCTTCAGGGGTCCTTAG
*Alp*	CCAACTCTTTTGTGCCAGAGA	GGCTACATTGGTGTTGAGCTTTT

**Figure 1.**
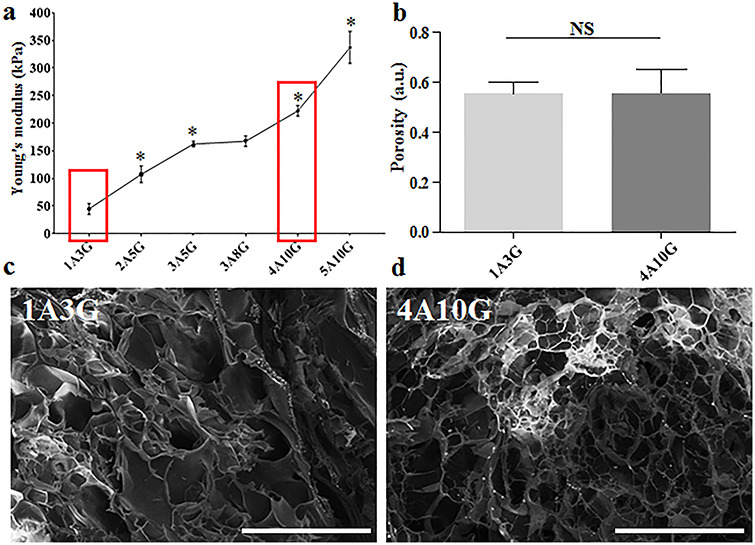
Construction of Alg-Gel composite hydrogel with different stiffness. (**a**) Alg-Gel composite hydrogels (1A3G, 2A5G, 3A5G, 3A8G, 4A10G and 5A10G) with different degrees of stiffness; ^*^ < 0.05, each compared with the previous group. (**b**) The porosity of 3D-bioprinted matrices 1A3G and 4A10G. (**c**, **d**) The microstructures of 3D-bioprinted matrices 1A3G and 4A10G observed using SEM; representative images selected (× 220 magnification, scale bar: 200 μm). Error bars represent standard deviation of the mean. *NS* not significant, *a.u.* arbitrary unit, *Alg-Gel* alginate-gelatin, *SEM* scanning electron microscope

### Co-staining with alkaline phosphatase and oil red O

The cells obtained after dissolution [32] of the matrices were resuspended in PBS. The cell suspension was evenly spread on the adherent glass slide, which was then dried at 60°C for 8 min. An oil red O staining kit (Solarbio, China) was used to stain adipocytes, while an alkaline phosphatase (ALP) staining kit (Solarbio, China) was used to stain osteoblasts. An optical microscope (Leica, DM2500, Germany) was used to obtain images.

### Statistical analysis

The data shown are expressed as mean ± standard deviation. IBM SPSS software 24 was used to perform the statistical analysis. Student’s ***t***-test was used to analyse the differences between two groups and one-way ANOVA post-hoc test was used to analyse the differences between multiple groups. The *p*-values < 0.05 were considered statistically significant.

## Results

### Fabrication of MSC-laden 3D-bioprinted Alg-Gel hydrogel matrices with different degrees of stiffness

In this experiment, we used Alg-Gel, which is commonly used in 3D bioprinting research, as the hydrogel matrix. Using different concentrations of sodium alginate and gelatin, six groups of Alg-Gel blends with different degrees of stiffness were constructed (1A3G, 2A5G, 3A5G, 3A8G, 4A10G, 5A10G). During the preparation process, we found that the 1A3G group had the lowest viscosity and the highest fluidity, while the 5A10G group had the highest viscosity and the worst fluidity at room temperature. Considering the operability of the subsequent experiments, the Alg-Gel blend with a higher concentration of alginate and gelatin was not used in this experiment. After crosslinking with 2.5% CaCl_2_, the Young’s modulus of the six groups of Alg-Gel composite hydrogels was measured, and the values are shown in Figure 1a (1A3G: 50 kPa, 2A5G: 110 kPa, 3A5G: 165 kPa, 3A8G: 165 kPa, 4A10G: 225 kPa, 5A10G: 355 kPa). Except for the 3A5G and 3A8G groups, significant differences were observed between the other two adjacent groups (Figure 1a). This indicated that the stiffness of the Alg-Gel composite hydrogels was primarily related to the concentration of sodium alginate and that stiffness was not significantly correlated with gelatin concentration. Since the cell suspension needed to be added to the hydrogels in subsequent experiments, the poor fluidity of the 5A10G group might have caused inadequate mixing of the hydrogel and the cell suspension. Therefore, we selected the 1A3G group and the 4A10G group, which had the largest differences in Young’s modulus, for subsequent experiments (Figure 1a red boxes). SEM images and porosity test results revealed no significant difference in the microstructure or porosity between the 1A3G group and the 4A10G group (Figure 1b, c and d).

**Figure 2.**
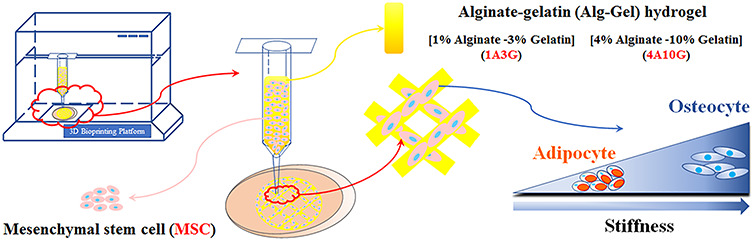
Schematic diagram of constructing the MSC-laden 3D-bioprinted matrices

1A3G and 4A10G hydrogels were used to generate the MSC-laden 3D-bioprinted matrices on the 3D bioprinting platform (Figure 2). During the entire culture period after printing, the composite hydrogels were cultured in DMEM or O/A medium. The media were changed once a day. The YAP inhibitors VP and GW5074 were dissolved in DMSO and added to the two media at working concentrations of 1 and 5 μM, respectively. DMSO without inhibitors was used as the control.

### Cell adhesion, extension and viability in 3D-bioprinted matrices

**Figure 3.**
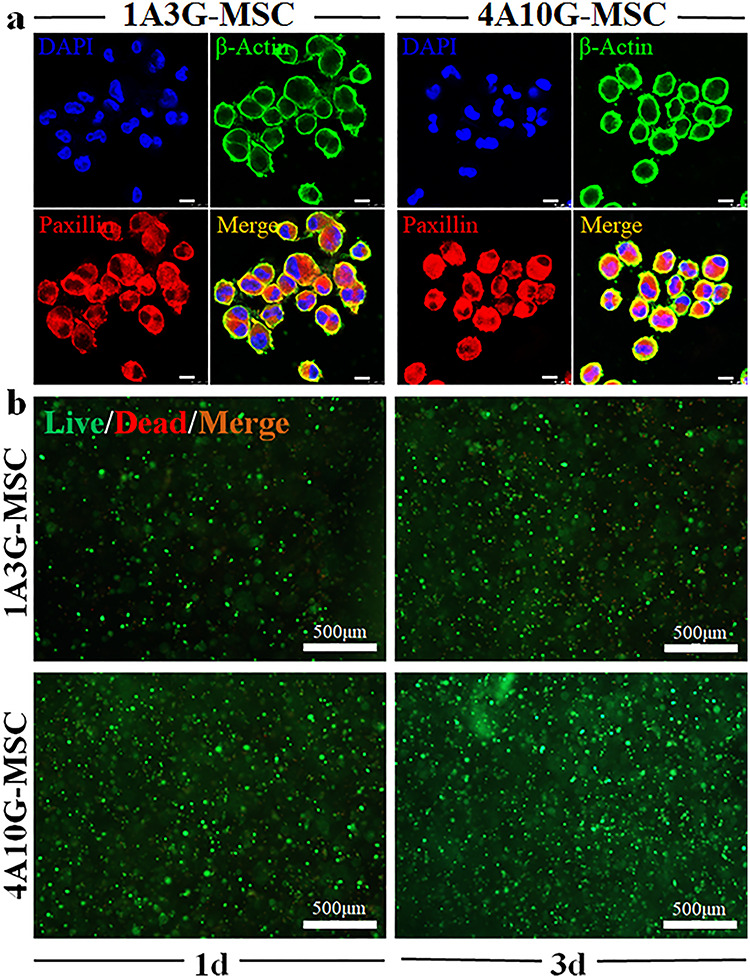
Cell adhesion, extension and viability of MSCs encapsulated in the 3D-bioprinted matrices. (**a**) Immunofluorescence staining for paxillin and β-actin in the 1A3G-MSC and 4A10G-MSC groups. Cell nuclei were stained with DAPI; representative images selected (× 640 magnification, scale bar: 8 μm). (**b**) Cell viability in the 1A3G-MSC and 4A10G-MSC groups. Live cells were stained with green fluorescence and dead cells were stained with red fluorescence; representative images selected (× 50 magnification, scale bar: 500 μm). *1d* day 1, *3d* day 3, *MSC* mesenchymal stem cell, *DAPI* 4', 6-diamidino-2-phenylindole

Good biocompatibility is an essential feature of hydrogels used in 3D bioprinting. In this experiment, immunofluorescence staining and cell viability tests were performed to determine the biocompatibility of the 1A3G and 4A10G hydrogels. Paxillin is a cytoskeletal protein involved in the attachment of an actin membrane at the site of extracellular stromal cell adhesion (focal adhesion). β-Actin is present at the extended edge of the cell and is an important cytoskeletal component. Therefore, on the first day after printing, immunofluorescence staining for paxillin and β-actin was performed to detect cell adhesion and expansion of the MSCs encapsulated in the 3D-bioprinted matrix. The fluorescence images showed no significant difference in cell extension or morphology between the 1A3G group and the 4A10G group (Figure 3a). At days 1 and 3 after printing, no significant difference in cell viability was observed between the 1A3G group and the 4A10G group (Figure 3b).

**Figure 4.**
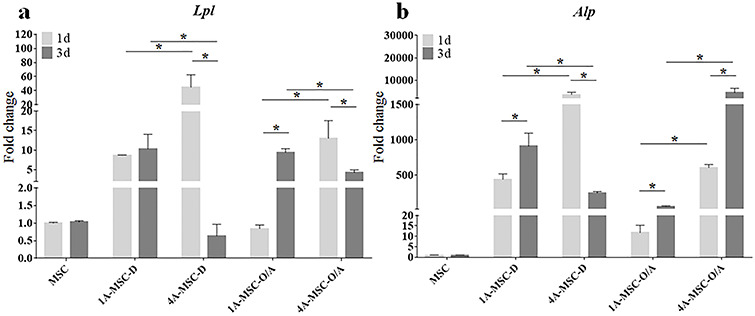
Gene expression histograms of the osteogenic and adipogenic differentiation of MSCs in 3D-bioprinted matrices. (**a**) The expression of *Lpl* in MSCs, the 1A3G (1A-MSC-D and 1A-MSC-O/A) and 4A10G (4A-MSC-D and 4A-MSC-O/A) groups were determined separately. (**b**) Expression of *Alp* in MSCs, the 1A3G (1A-MSC-D and 1A-MSC-O/A) and 4A10G (4A-MSC-D and 4A-MSC-O/A) groups were determined separately. Gene expression was normalized relative to *Gapdh* expression. ^*^p < 0.05. Error bars represent standard deviation of the mean. *1d* day 1, *3d* day 3, *MSC* mesenchymal stem cell, *D* DMEM, *O/A* O/A medium, *Alp* alkaline phosphatase, *Lpl* lipoprotein lipase, *Gapdh* glyceraldehyde 3-phosphate dehydrogenase

### Osteogenic and adipogenic differentiation of MSCs in 3D-bioprinted matrices

DMEM and O/A media were used to culture the 3D-bioprinted matrices consisting of the hydrogels from groups 1A3G and 4A10G. The experimental groups cultured with DMEM were labelled ‘1A-MSC-D’ and ‘4A-MSC-D’, while the experimental groups cultured with O/A medium were labelled ‘1A-MSC-O/A’ and ‘4A-MSC-O/A’. ALP is a specific group of phosphatases secreted by osteoblasts, and lipoprotein lipase (LPL), the core enzyme in lipid metabolism, is a glycoprotein synthesized and secreted by adipocytes. Therefore, PCR was used to detect the expression levels of *Lpl* and *Alp* in the experimental groups.

In the DMEM group, on day 1, the expression levels of *Lpl* and *Alp* in the 4A-MSC-D group were significantly higher than those in the 1A-MSC-D group (Figure 4a and b). After the time window of mechanosensitivity had passed [33], the ‘stiffness-effect’ seemed to be weakened. By day 3, the expression of *Lpl* did not change significantly in the 1A-MSC-D group, but its expression was significantly decreased in the 4A-MSC-D group (Figure 4a). On day 3, the expression of *Alp* was increased in the 1A-MSC-D group, but significantly decreased in the 4A-MSC-D group (Figure 4b).

In the O/A medium group, the expression levels of *Lpl* and *Alp* in the two groups showed different trends compared with the DMEM group. On day 1, the expression levels of *Lpl* and *Alp* in the 4A-MSC-O/A group were significantly higher than those in the 1A-MSC-O/A group (Figure 4a and b). On day 3 after printing, the expression of *Lpl* was significantly increased in the 1A-MSC-O/A group and was significantly decreased in the 4A-MSC-O/A group, which indicated that *Lpl* expression was significantly higher in the 1A-MSC-O/A group at day 3 compared with the 4A-MSC-O/A group (Figure 4a). On day 3, the expression of *Alp* was significantly increased in both the 1A-MSC-O/A and 4A-MSC-O/A groups (Figure 4b). However, the increase in *Alp* expression in the 4A-MSC-O/A group was much higher than that in the 1A-MSC-O/A group, which suggested that *Alp* expression was significantly higher in the 4A-MSC-O/A than in the 1A-MSC-O/A group (Figure 4b). Although this ‘stiffness-effect’ could stimulate the strong tendency of MSCs to differentiate into osteoblasts and adipocytes independently of inductive factors, the induction was only an initial effect, especially for stiffer matrices.

**Figure 5.**
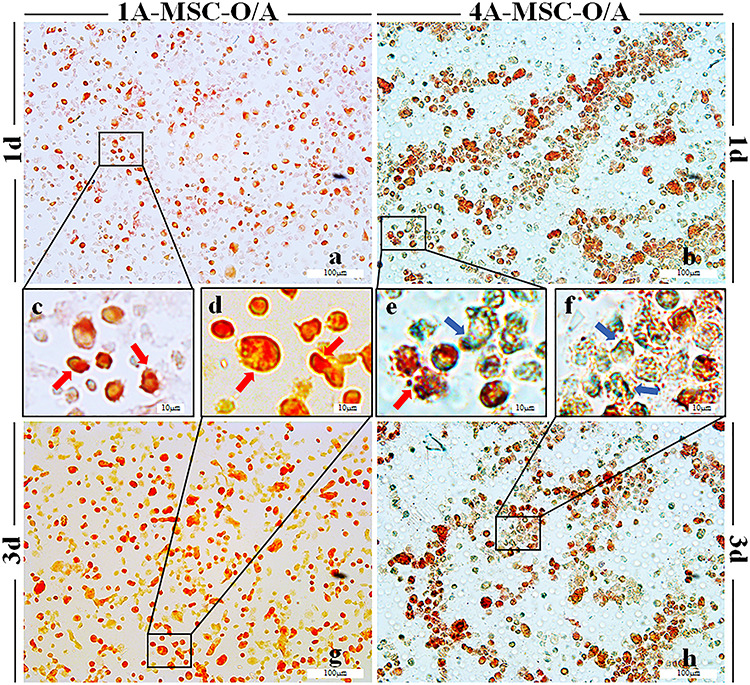
Histological staining of MSCs in 3D-bioprinted matrices. (a, c, d, g) Co-staining with ALP and oil red O in the 1A-MSC-O/A group on day 1 (a, c) and day 3 (d, g). a, g × 50 magnification, scale bar: 100 μm; c, d × 265 magnification, scale bar: 10 μm. (b, e, f, h) Co-staining with ALP and oil red O in the 4A-MSC-O/A group on day 1 (b, e) and day 3 (f, h). b, h × 50 magnification, scale bar: 100 μm; e, f × 265 magnification, scale bar: 10 μm. The red arrows (c, d, e) indicate oil red O-stained adipocytes, and the blue arrows (e, f) indicate ALP-stained osteoblasts (a, b, g, h × 50 magnification, scale bar: 100 μm; c, d, e, f × 265 magnification, scale bar: 10 μm). *1d* day 1, *3d* day 3, *MSC* mesenchymal stem cell, *O/A* O/A medium, *ALP* alkaline phosphatase

The results of the O/A medium group were also confirmed by co-staining with ALP and oil red O (Figure 5). At days 1 and 3, MSCs encapsulated in group 1A-MSC-O/A hydrogels largely differentiated into adipocytes (Figure 5a, c, d and g). In the 4A-MSC-O/A group, the encapsulated MSCs differentiated largely into osteoblasts and adipocytes by day 1. Moreover, the MSCs largely differentiated into osteoblasts by day 3 (Figure 5b, e, f, h).

### Inhibition of YAP in the osteogenic and adipogenic differentiation of MSCs without inductive factors

Several studies have shown that matrix stiffness affected MSC differentiation through intracellular mechanical force transduction regulated by YAP and its mechanical signalling pathways. To explore whether matrix stiffness affected osteogenic and adipogenic differentiation of MSCs independently of biochemical factors, YAP inhibitors (VP and GW5074) were added to the DMEM, and the experimental groups were labelled accordingly: 1A-MSC-D, 1A-MSC-D-Control, 1A-MSC-D-VP, 1A-MSC-D-GW5074, 4A-MSC-D, 4A-MSC-D-Control, 4A-MSC-D-VP and 4A-MSC-D-GW5074. However, VP and GW5074 are two different types of inhibitors, and thus, the sites and modes of action of their inhibitory mechanisms on YAP are different, which in turn contributes to the difference in their inhibitory experimental effects [34].

**Figure 6.**
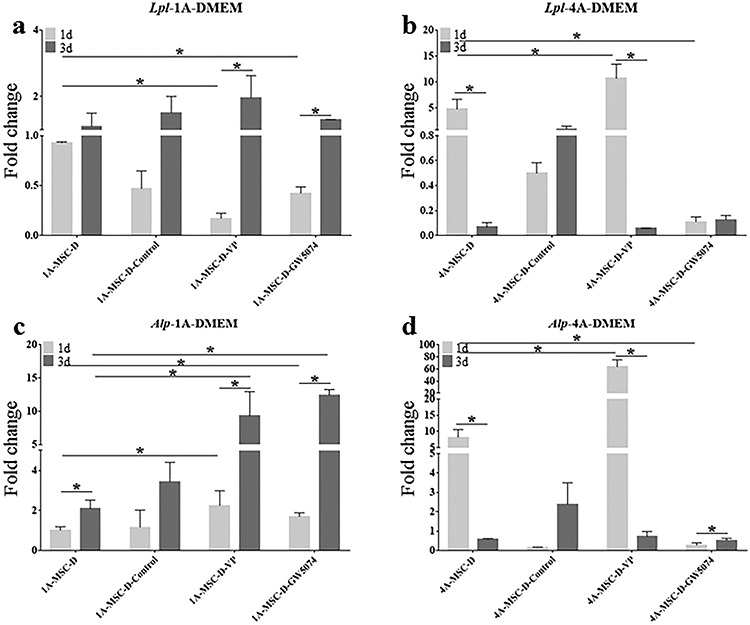
Gene expression histograms of inhibition of YAP in the osteogenic and adipogenic differentiation of MSCs without inductive factors. Expression levels of *Lpl* (**a**) and *Alp* (**c**) in the 1A3G group cultured with the DMEM (1A-MSC-D, 1A-MSC-D-Control, 1A-MSC-D-VP and 1A-MSC-D-GW5074) were determined separately. Expression levels of *Lpl* (**b**) and *Alp* (**d**) in the 4A10G group cultured with the DMEM (4A-MSC-D, 4A-MSC-D-Control, 4A-MSC-D-VP and 4A-MSC-D-GW5074) were determined separately. Gene expression was normalized relative to *Gapdh* expression. ^*^p < 0.05. Error bars represent standard deviation of the mean. *1d* day 1, *3d* day 3, *MSC* mesenchymal stem cell, *D* DMEM, *Alp* alkaline phosphatase, *Lpl* lipoprotein lipase, *Gapdh* glyceraldehyde 3-phosphate dehydrogenase

On day 1, the expression of *Lpl* was significantly lower in the 1A-MSC-D-VP and 1A-MSC-D-GW5074 groups than in the 1A-MSC-D group (Figure 6a). By day 3, the expression of *Lpl* in the 1A-MSC-D-VP and 1A-MSC-D-GW5074 groups was significantly increased, but its expression was not significantly different from that in the 1A-MSC-D group. On day 1, the expression of *Lpl* in the 4A-MSC-D-VP group was significantly higher than that in the 4A-MSC-D group, while the expression of *Lpl* in the 4A-MSC-D-GW5074 group was significantly lower than that in the 4A-MSC-D group (Figure 6b). By day 3, the expression of *Lpl* in the 4A-MSC-D-VP and 4A-MSC-D groups was significantly decreased. The expression of *Lpl* in the 4A-MSC-D-VP and 4A-MSC-D-GW5074 groups was not significantly different from that in the 4A-MSC-D group. No significant difference was found in *Lpl* expression between the 4A-MSC-D-VP group and the 4A-MSC-D group or between the 4A-MSC-D-GW5074 group and the 4A-MSC-D group (*p* > 0.05).

**Figure 7.**
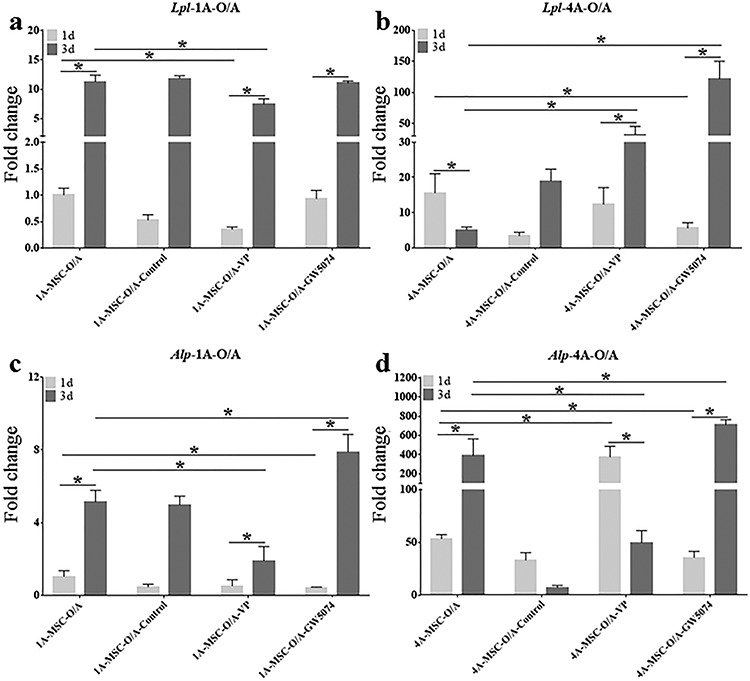
Gene expression histograms of inhibition of YAP in the osteogenic and adipogenic differentiation of MSCs. Expression levels of *Lpl* (**a**) and *Alp* (**c**) in the 1A3G group cultured with O/A medium (1A-MSC-O/A, 1A-MSC-O/A-Control, 1A-MSC-O/A-VP and 1A-MSC-O/A-GW5074) were determined separately. Expression levels of *Lpl* (**b**) and *Alp* (**d**) in the 4A10G group cultured with O/A medium (4A-MSC-O/A, 4A-MSC-O/A-Control, 4A-MSC-O/A-VP and 4A-MSC-O/A-GW5074) were determined separately. Gene expression was normalized relative to *Gapdh* expression. ^*^p < 0.05. Error bars represent standard deviation of the mean. *1d* day 1, *3d* day 3, *MSC* mesenchymal stem cell, *O/A* O/A medium, *Alp* alkaline phosphatase, *Lpl* lipoprotein lipase, *Gapdh* glyceraldehyde 3-phosphate dehydrogenase

**Figure 8.**
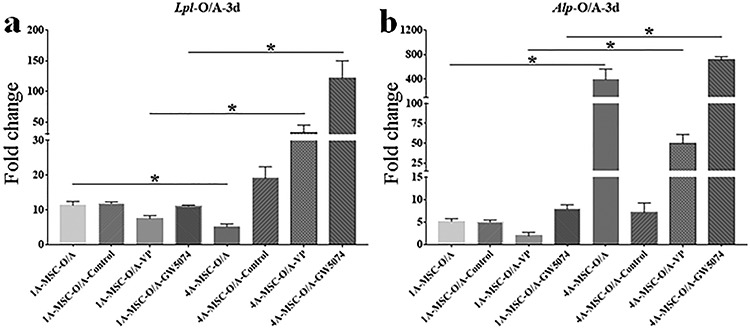
Gene expression histograms of inhibition of YAP in the osteogenic and adipogenic differentiation of MSCs on day 3. (**a**) Expression of *Lpl* in the 1A3G group cultured with O/A medium (1A-MSC-O/A, 1A-MSC-O/A-Control, 1A-MSC-O/A-VP and 1A-MSC-O/A-GW5074) and the 4A10G group cultured with O/A medium (4A-MSC-O/A, 4A-MSC-O/A-Control, 4A-MSC-O/A-VP and 4A-MSC-O/A-GW5074) on day 3. (**b**) Expression of *Alp* in the 1A3G group cultured with O/A medium (1A-MSC-O/A, 1A-MSC-O/A-Control, 1A-MSC-O/A-VP and 1A-MSC-O/A-GW5074) and the 4A10G group cultured with O/A medium (4A-MSC-O/A, 4A-MSC-O/A-Control, 4A-MSC-O/A-VP and 4A-MSC-O/A-GW5074) on day 3. Gene expression was normalized relative to *Gapdh* expression. ^*^p < 0.05. Error bars represent standard deviation of the mean. *3d* day 3, *MSC* mesenchymal stem cell, *O/A* O/A medium, *Alp* alkaline phosphatase, *Lpl* lipoprotein lipase, *Gapdh* glyceraldehyde 3-phosphate dehydrogenase

On day 1, the expression of *Alp* in the 1A-MSC-D-VP and 1A-MSC-D-GW5074 groups was slightly higher than that in the 1A-MSC-D group (Figure 6c). By day 3, the expression of *Alp* in the 1A-MSC-D-VP and 1A-MSC-D-GW5074 groups was significantly increased so that the expression of *Alp* in those two groups was significantly higher than that in the 1A-MSC-D group. On day 1, the expression of *Alp* in the 4A-MSC-D-VP group was significantly higher than that in the 4A-MSC-D group, while the expression of *Alp* in the 4A-MSC-D-GW5074 group was significantly lower than that in the 4A-MSC-D group (Figure 6d). By day 3, the expression of *Alp* in the 4A-MSC-D-GW5074 group was slightly increased. However, the expression of *Alp* in the 4A-MSC-D-VP and 4A-MSC-D-GW5074 groups was not significantly different from that in the 4A-MSC-D group.

The above results indicated that matrix stiffness exerted a short-term strong and positive effect on the osteogenic and adipogenic differentiation of MSCs. However, due to the lack of osteogenic- and adipogenic-inducing media, the induction effect of matrix stiffness could not be maintained for a long period of time. Therefore, in order to investigate the mechanism by which matrix stiffness regulates the osteogenic and adipogenic differentiation of MSCs, we added two YAP inhibitors to the O/A medium-treated groups in the following experiment.

### Inhibition of YAP in the osteogenic and adipogenic differentiation of MSCs

YAP inhibitors (VP and GW5074) were added to the O/A medium-treated groups. The groups in this experiment were 1A-MSC-O/A, 1A-MSC-O/A-Control, 1A-MSC-O/A-VP, 1A-MSC-O/A-GW5074, 4A-MSC-O/A, 4A-MSC-O/A-Control, 4A-MSC-O/A-VP and 4A-MSC-O/A-GW5074.

On day 1, the expression of *Lpl* in the 1A-MSC-O/A-VP and 1A-MSC-O/A-GW5074 groups was slightly lower than that in the 1A-MSC-O/A group (Figure 7a). By day 3, the expression of *Lpl* in the 1A-MSC-O/A, 1A-MSC-O/A-VP and 1A-MSC-O/A-GW5074 groups was significantly increased. However, the expression of *Lpl* in the 1A-MSC-O/A-VP group was significantly lower than that in the 1A-MSC-O/A group. Moreover, the expression of *Lpl* in the 1A-MSC-O/A-GW5074 group was not significantly different from that in the 1A-MSC-O/A group. On day 1, the expression of *Lpl* in the 4A-MSC-O/A-VP group was not significantly different from that in the 4A-MSC-O/A group, while the expression of *Lpl* in the 4A-MSC-O/A-GW5074 group was significantly lower than that in the 4A-MSC-O/A group (Figure 7b). By day 3, the expression of *Lpl* in the 4A-MSC-O/A-VP and 4A-MSC-O/A-GW5074 groups was significantly increased. The expression of *Lpl* in the 4A-MSC-O/A-VP and 4A-MSC-O/A-GW5074 groups was significantly higher than that in the 4A-MSC-O/A group.

On day 1, the expression of *Alp* in the 1A-MSC-O/A-VP group was not significantly different from that in the 1A-MSC-O/A group, while the expression of *Alp* in the 1A-MSC-O/A-GW5074 group was slightly lower than that in the 1A-MSC-O/A group (Figure 7c). By day 3, the expression of *Alp* in the 1A-MSC-O/A-VP and 1A-MSC-O/A-GW5074 groups was significantly increased. The expression of *Alp* in the 1A-MSC-O/A-VP group was slightly lower than that in the 1A-MSC-O/A group, while the expression of *Alp* in the 1A-MSC-O/A-GW5074 group was slightly higher than that in the 1A-MSC-O/A group. On day 1, the expression of *Alp* in the 4A-MSC-O/A-VP group was significantly higher than that in the 4A-MSC-O/A group, while the expression of *Alp* in the 4A-MSC-O/A-GW5074 group was slightly lower than that in the 4A-MSC-O/A group (Figure 7d). By day 3, the expression of *Alp* in the 4A-MSC-O/A-VP group was significantly decreased, which indicated that *Alp* expression in the 4A-MSC-O/A-VP group was significantly lower than in the 4A-MSC-O/A group. The expression of *Alp* in the 4A-MSC-O/A-GW5074 group was significantly increased on day 3, but the expression of *Alp* in the 4A-MSC-O/A-GW5074 group was only slightly higher than that in the 4A-MSC-O/A group. Overall, with O/A medium, the expression levels of *Lpl* and *Alp* in the 4A10G group was significantly higher than that in the 1A3G group treated with O/A medium by 3 days after printing (Figure 8a and b).

## Discussion

The cellular microenvironment can affect MSC differentiation into various types of adult cells, such as osteoblasts, adipocytes and neuroblasts, through the synergistic effects of both biochemical and biophysical cues [22–25]. For example, several studies have shown that MSCs encapsulated within 3D matrices with tuneable stiffness can initiate osteogenic and adipogenic differentiation programmes [24, 35, 36]. Although biophysical cues control MSC lineage specification probably via cytoskeletal reorganization, the complete mechanism is largely unclear. It is also unknown whether physical effects could act independently of biochemical inducers [24, 35, 37–39]. In this study, with a focus on understanding the interplay between physical cues and cell fate determination of MSCs, we found that hydrogel-based bioink stiffness could individually contribute to the osteogenic and adipogenic differentiation of MSCs in 3D-printed matrices. More importantly, biophysical cues such as stiffness might initiate or strengthen the biochemical signaling involved in MSC fate determination and differentiation.

Based on our previous study, we formulated two types of Alg-Gel composite hydrogels with different ratios (1A3G vs. 4A10G) and different stiffness values (Young’s modulus: 50 vs. 225 kPa). Recently, some studies on the fate of MSCs encapsulated within 3D-bioprinted matrices have proposed that, in addition to biological and structural cues [5, 6, 8, 40], the pore size and porosity [41–44] also regulate stem cell differentiation within Alg-Gel hydrogel-based bioinks [9, 11–14]. Therefore, we constructed these Alg-Gel composite hydrogels with different degrees of stiffness without changing their pore size and porosity so that we could study the effect of matrix stiffness independently of other biophysical cues on cell fate decisions. SEM analysis showed that the 1A3G and 4A10G hydrogel groups selected in this experiment only exhibited significant differences in stiffness but exhibited almost no differences in porosity and micropore structure. In addition, by evaluating cell extension, cell adhesion and cell viability of MSCs encapsulated in the 3D-bioprinted matrices, we also confirmed that the different stiffness values between the 1A3G and 4A10G groups did not result in differences in cell extension, cell adhesion or cell viability.

To eliminate the effects of extrinsic inductive factors, we cultivated the 3D-bioprinted matrices composed of the 1A3G and 4A10G hydrogels in two media (DMEM and O/A medium). The PCR results showed that the 4A10G group, which was stiffer, exhibited significant upregulation of *Lpl* and *Alp* expression 1 day after printing without inductive factors, but this phenomenon significantly decreased at 3 days. With the presence of inductive factors, the stiffer 4A10G group also demonstrated a significant increase in *Lpl* and *Alp* expression 1 day after printing. However, until day 3, the stiffer 4A10G group exhibited a more significant increase in *Alp* and *Lpl* expression upon the addition of inductive factors, while the softer 1A3G group exhibited a more significant increase in *Lpl* expression with inductive factors. The softer matrix induces the adipogenic differentiation of MSCs, while the stiffer matrix induces the osteogenic differentiation of MSCs, which is similar to what has been reported in previous studies [24, 35, 37]. In addition, co-staining with ALP and oil red O also confirmed the PCR results. The MSCs encapsulated in the 1A3G-MSC-O/A group tended to differentiate into adipocytes, while the MSCs encapsulated within the 4A10G-MSC-O/A group tended to differentiate into osteoblasts during the culture period. These results indicated that matrix stiffness could stimulate the stronger tendency of MSCs to differentiate into osteoblasts and adipocytes independently of inductive factors but that the inductive effect of matrix stiffness might be exhibited only during the early stage. Moreover, these results implied that the stiffness of the hydrogel itself (with no differentiation medium) has an obvious effect on differentiation during the early stage, while this ‘stiffness-effect’ seemed to gradually become enervated or compromised by the biochemical effect of the culture medium.

Stiffness-driven stem cell differentiation is probably directed by mechanotransduction effects, as stem cells are usually mechanosensitive at the initiation of differentiation [33]. The mechanotransduction effects can further affect intracellular and intercellular signalling pathways and ultimately result in stiffness-regulated stem cell behaviour [36, 37]. YAP is a key protein in the Hippo pathway of mechano-transduction [45], and many studies have shown that the mechano-transduction triggered by matrix stiffness in stem cells is mediated by YAP [26, 27, 46, 47]. Therefore, we further selected two YAP inhibitors (VP and GW5074) to verify whether the effect of matrix stiffness on the osteogenic and adipogenic differentiation of MSCs was transduced by YAP. Cells in the 1A3G group without inductive factors exhibited a slight upregulation in *Alp* expression after YAP inhibition, while cells in the 4A10G group without inductive factors exhibited a slight upregulation in *Lpl* expression only on day 1 after YAP inhibition. When the matrices were cultured with inductive factors, only the 4A10G group exhibited significant upregulation of *Lpl* expression and downregulation of *Alp* expression when YAP was inhibited.

However, at some time points, the groups in which YAP was inhibited seemed to show potent differentiation. We speculated that three main reasons might be responsible for these trends. First, VP and GW5074 are two different types of inhibitors, and thus, their sites and the modes of action of their inhibitory mechanisms on YAP are different, which in turn contributes to the difference in their inhibitory experimental effects [34]. Second, although this ‘stiffness-effect’ could stimulate the strong tendency of MSCs to differentiate into osteoblasts and adipocytes independently of inductive factors, the inductive effect was only observed during the early stage. Third, YAP expression is closely correlated with the mechanosensitivity of stem cells early in differentiation, and other regulatory effects may be able to override the effect of mechanosensitivity by manipulating YAP expression at other time points throughout the lineage commitment process; alternatively, stiffness might drive other compensatory YAP pathways. Although we did not investigate other possible signalling pathways in this study, it was observed that MSCs might sense mechanical alterations in the ECM and respond to mechanotransduction effects, which subsequently dictate their commitment to a specific lineage.

## Conclusion

Taken together, these results provide evidence for the effects of hydrogel-based bioink stiffness on MSC differentiation. More importantly, stiffness might initiate or strengthen the biochemical signalling involved in MSC fate determination and differentiation. Although the regulating mechanism of MSC differentiation is complex and can be multifaceted, the results from this study shed some light on the importance of future studies on the central and functional role of biophysical cues during cell lineage commitment in 3D bioprinting.

## Abbreviations

Alg-Gel: alginate–gelatin; ALP: alkaline phosphatase; DMEM: Dulbecco’s modified Eagle’s medium; DMSO, dimethyl sulfoxide: ECM: extracellular matrix; LPL: lipoprotein lipase; MSC: mesenchymal stem cell; O/A medium: osteogenic- and adipogenic-inducing medium; PCR: polymerase chain reaction; VP, Verteporfin; YAP: Yes-associated protein.

## Funding

This study was supported in part by the National Nature Science Foundation of China (81830064, 81721092 and 81701906), the National Key Research and Development Plan (2017YFC1103300), Funds Chinese PLA General Hospital for Military Medical Innovation Research Project (CX19026), the CAMS Innovation Fund for Medical Sciences (CIFMS, 2019-I2M-5-059) and the Military Medical Research and Development Projects (AWS17J005, 2019–126) and Fostering Funds of Chinese PLA General Hospital for National Distinguished Young Scholar Science Fund (2017-JQPY-002).

## Availability of data and materials

The authors declare that the main data supporting the findings of this study are available within the article. Extra data are available from the corresponding author upon request.

## Authors’ contributions

YL was a major contributor in writing the manuscript. YL, ZL, JL, SY, YZ, BY and WS performed the experiments and analysed the data. XF and SH supervised the writing of the manuscript and revised it critically for important intellectual content. All authors read and approved the final manuscript.

## Conflict of interest

The authors declare no conflict of interest.
